# Novel Insights Into Pathogenesis and Therapeutic Strategies of Hepatic Encephalopathy, From the Gut Microbiota Perspective

**DOI:** 10.3389/fcimb.2021.586427

**Published:** 2021-02-22

**Authors:** Jiachen Liu, Yantao Xu, Bimei Jiang

**Affiliations:** ^1^ Xiangya Medical College of Central South University, Changsha, China; ^2^ Department of Pathophysiology, Sepsis Translational Medicine Key Laboratory of Hunan Province, Xiangya School of Medicine, Central South University, Changsha, China

**Keywords:** gut microbiota, hepatic encephalopathy, gut–liver–brain axis, probiotic, fecal microbiota transplantation, antibiotics

## Abstract

Since the 1950s, gradual changes in the gut microbiota of patients with hepatic encephalopathy have been observed. Previous research has indicated potential associations between the gut and brain, and the gut microbiota is becoming a hot topic in research on diseases of the nervous system. However, for the past few decades, studies of hepatic encephalopathy have been restricted to controlling the gut microbiota during macroscopic manipulation, such as probiotic intervention, while its clinical use remains controversial, and the cellular mechanisms underlying this condition are still poorly understood. This thesis seeks to comprehensively understand and explain the role of gut microbiota in hepatic encephalopathy as well as analyze the effects of intervention by regulating the gut microbiota. Evidence is presented that shows that dysbiosis of the gut microbiota is the primary pathological driver of hepatic encephalopathy and impacts pathologic progression *via* complex regulatory networks. As a result, suggestions were identified for future mechanistic research and improvements in therapeutic strategies for hepatic encephalopathy.

## Highlights

Pathogenesis of hepatic encephalopathy are summarized from gut microbiota perspective.

- Intervention effects of hepatic encephalopathy *via* regulating the gut microbiota are analyzed.
- Novel perspectives on therapeutic strategy of hepatic encephalopathy are introduced.

## Introduction

Hepatic encephalopathy (HE) is one of the most severe complications of cirrhosis and involves alteration of consciousness, which is directly related to liver failure (Bajaj, 2018). According to the latest guidelines published in 2018, HE is defined as brain dysfunction caused by liver failure and/or portal-systemic blood shunting that produces a spectrum of neurological/psychiatric abnormalities ranging from subclinical alterations to coma (Montagnese et al., 2019). HE is still indisputably associated with hepatic insufficiency; therefore, it is reasonable to characterize this encephalopathy as HE (Yanny et al., 2019). According to epidemiological investigations, the prevalence of HE is approximately 30 to 45%, which is accompanied by an increase in mortality (Patidar and Bajaj, 2015; Xu et al., 2019). In the United States, the economic burden of hospitalization for HE amounts to more than $11.9 billion each year (Hirode et al., 2019), which causes significant increases in patient mobility, morbidity, and care utilization (Jang et al., 2020).

**Figure 1:**
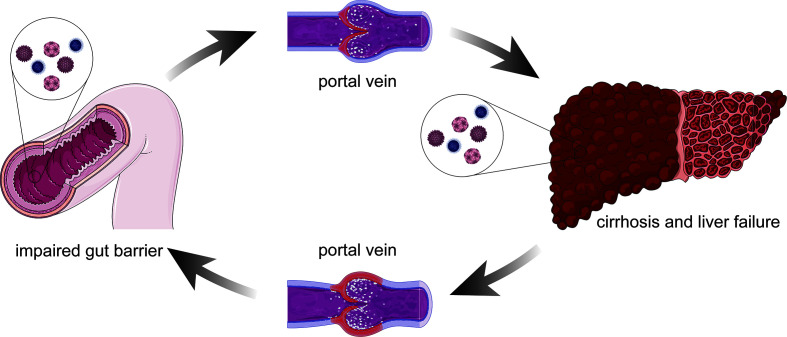
Association between the damaged intestinal mucosa liver failure. Altered immune function such as impaired gut barrier, bacterial products can reach the liver through the portal vein and might lead to a pro-inflammatory response. The pro-inflammatory stimulus facilitates hepatocyte damage and central process that promotes liver inflammation. On the other hand, the defunctionalization of the liver carries on impairing the gut barrier due to the portal hypertension. Left untreated, it can lead to cirrhosis and liver failure.

The number of microorganisms inhabiting the gut (the largest surface area in the body) has been estimated to exceed 100 quintillion (Biver et al., 2019). In the local immune system, the gut epithelium is a major line of defense in which epithelial cells provide a physical barrier and work in concert with immune and stromal cells to fight off microbes and toxins. Increasing evidence suggests that gut barrier dysfunction can lead to liver failure, which can in turn aggravate intestinal mucosal injury (Arab et al., 2018; Xu et al., 2018) (**Figure 1**). During chronic liver failure, ammonia accumulation in the brain leads to Alzheimer type 2 astrocytosis, which has been highlighted as neuropathological characteristics of HE (Butterworth, 2019). Besides, maintained inflammation caused by hyperammonemias will promote the development of systemic and central inflammation (Aitbaev et al., 2017), which will cause activation of the microglia indicative of neuroinflammation (Rodrigo et al., 2010). Therefore, there is a potential but important interaction between the gut microbiota and HE, which will provide a novel perspective on HE and inform the development of future interventions (Bajaj, 2019).

## HE and Microbiota: Interaction

The key to understanding the pathogenesis of HE is to adequately describe the physiological fluctuations and pathological shifts of the gut–brain–liver axis as well as gut microbiota metabolic interactions. A more detailed account of the pathological process is given in the following section.

### The Role of the Gut–Brain–Liver Axis in the Pathogenesis of HE

**Figure 2:**
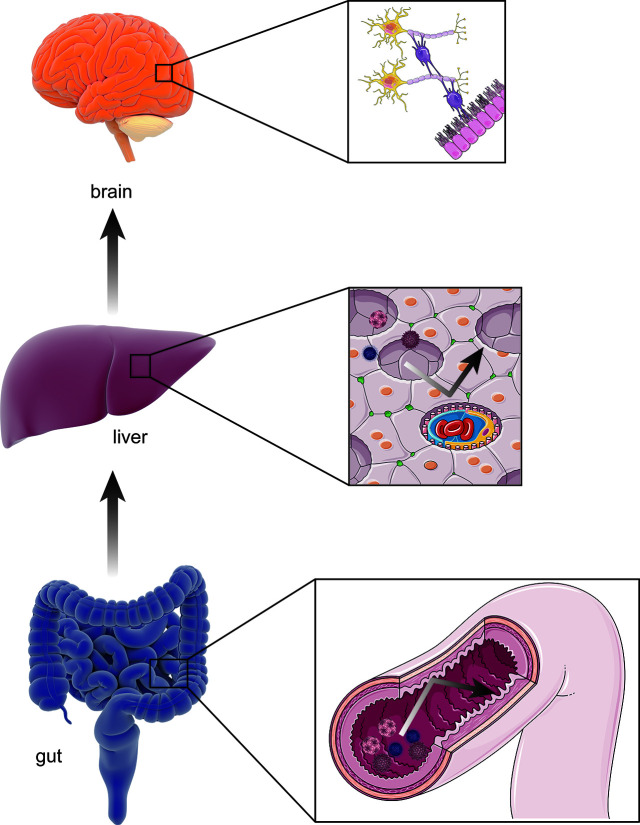
The healthy gut–liver–brain axis. A healthy gut**–**liver**–**brain axis contains gut and its microbiota, the liver, and the brain. In the normal condition, two “shields” including homeostasis of intestinal permeability as physical barrier and the normal hepatic function of amino-metabolism prevent potential pathogens and gut enteroendotoxin from hamming other organs (such as brain) through blood stream.

The gut**–**liver**–**brain axis refers to the bidirectional relationship between the gut and its microbiota, the liver, and the brain, resulting from the integration of signals generated by dietary, genetic, and environmental factors (Wiest et al., 2017; Mancini et al., 2018). A healthy, homeostatic condition of the gut–liver–brain axis provides two shields for the brain against the hazardous contents in the gut: one is the homeostasis of gut permeability, and the other is normal hepatic function (Mancini et al., 2018) (**Figure 2**). The gut is open to the external environment, resulting in a convenient pathway for microbiota colonization, and can be easily affected by changes in the environment. In addition, the gut is responsible for digestion and absorption, indicating the need for a strong permeability function to prevent any toxins in the gut from reaching the capillaries. However, the liver is a recipient and filter of nutrients, bacterial products, toxins and metabolites, both endogenous an exogenous (Wiest et al., 2017). The collapse of either shield causes several mutually reinforced abnormal states and leads to diseases such as cirrhosis, portal hypertension, and HE.

As a late complication of cirrhosis, HE is considered to be a typical disease owing to abnormal gut**–**liver**–**brain function, but the understanding of its pathogenesis is poor. Increasing evidence shows that alteration in the structure of the gut microbiota and its metabolic by-products, as well as a background of local and systemic inflammation and leaky gut, drive the development of HE (Azhari and Swain, 2018; Manzhalii et al., 2019; Jaffe et al., 2020).

**Figure 3:**
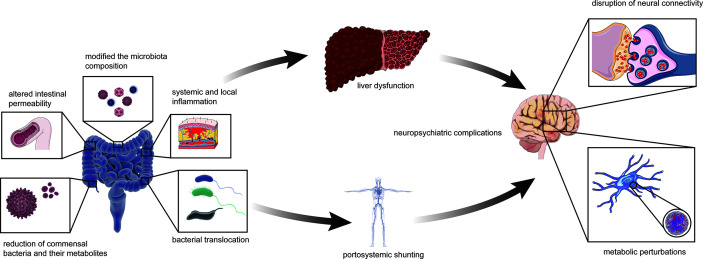
The collapsed gut–liver–brain in HE. The initial factor of gut microbiota dysbiosis can be liver disease possibly with relevant or irrelevant changes of intestinal barrier and portal system. Altered intestinal permeability, bacterial translocation, systemic and local inflammation, modified the microbiota composition, with reduction of commensal bacteria and their metabolites such as short-chain fatty acids, excessive amino and gamma-aminobutyric acid. The abnormal status finally affects liver function such as ammonia metabolism. Besides, liver disease can also cause portal-system shunt and let ammonia escape from the liver. Therefore, ammonia entering the body circulation and then the blood brain barrier, leading to neuropsychiatric complications such as hepatic encephalopathy.

The relationship between the gut–liver**–**brain axis and liver disease are likely to be described as a “chicken or egg” problem (Arab et al., 2018). The collapse of functions of the gut barrier is due to various causes, such as local infection and bacterial translocation, which can alter gut permeability, bacterial translocation, and systemic and local inflammation; modify the microbiota composition; and reduce commensal bacteria and their metabolites, such as short-chain fatty acids (SCFAs) (Aguirre Valadez et al., 2016; Fukui and Wiest, 2016). This phenomenon provides a condition for enteric endotoxins and harmful metabolites to enter the blood through the gut mucosa, thus damaging liver and brain functions as well as causing HE, which might be the initiating factor (Aguirre Valadez et al., 2016; Fukui and Wiest, 2016) (**Figure 3**).

However, liver function also affects the barrier function of the gut. Anatomically, the liver and gut are connected by the portal vein system (Covey et al., 2004). The normal functioning of the portal system as well as normal liver function allow harmful metabolic substances produced in the gut to be decomposed in the liver, thus maintaining the remaining part of the blood with normal levels of toxins (Wiest et al., 2017). Regulation of ammonia levels entering the portal circulation depends on the urea cycle in the liver as well as on muscle and kidney metabolism (Milosevic et al., 2019; Raj et al., 2020). Unfortunately, the loss of normal liver function and abnormal conditions, such as portal-systemic syndrome during the disease, reduce metabolic function. In cirrhosis, the increase of plasma ammonia in cirrhosis is mainly due to portal-systemic shunting, since ammonia is a substance highly extracted by the liver. Ammonia enters the circulation and then penetrates the blood brain barrier, leading to neuropsychiatric complications such as HE (Milosevic et al., 2019).

### The Role of Gut Microbiota in the Pathogenesis of HE From the Gut–Brain–Liver Axis Perspective

Although the pathogenesis of HE is unclear, the hypothesis that the microbiota plays an important role in this condition has been confirmed. Some evidence shows that the higher risk of microbiota dysbiosis in cirrhotic patients, with the subsequent clinical implications, is principally due to the various pathological interactions between the liver and the gastrointestinal tract. The alteration in gut motility, the higher pH, and the reduced bile acid concentrations in the colons of patients with cirrhosis may lead to a failure to control gut bacterial overgrowth.

In addition, cirrhosis impairs the homeostatic role of the liver in the systemic immune response. Damage to the reticuloendothelial system compromises the liver immune surveillance function exerted by Kupffer cells and sinusoidal endothelial cells, and the reduced hepatic synthesis of proteins involved in innate immunity and pattern recognition hinders the bactericidal ability of phagocytic cells (Katz et al., 1991; Sipeki et al., 2014). Monocyte spreading, chemotaxis, and neutrophil activity are also significantly reduced in patients with cirrhosis compared with controls (Fiuza et al., 2000). This phenomenon can in turn compromise the gut barrier and facilitate bacterial translocation, an increased risk of gut bacterial infections and decompensated liver disease (Schnabl and Brenner, 2014; Betrapally et al., 2017).

The bacterial composition of the sigmoid colon in patients with HE was significantly altered (Bajaj et al., 2012b). Recent studies have demonstrated lower *Roseburia* and higher *Enterococcus*, *Veillonella*, *Megasphaera*, and *Burkholderia* among sigmoid colonic mucosal microbiota in HE groups compared to controls. Moreover, genera including *Blautia*, *Fecalibacterium*, *Roseburia*, and *Dorea* can be considered biomarkers of cognition and inflammation that are related to acetylcholinesterase inhibitors (Bajaj et al., 2012a; Zhang et al., 2013). In addition, Bajaj and his colleagues demonstrated that Enterobacteriaceae, Fusobacteriaceae, and Veillonellaceae were positively and Ruminococcaceae negatively related to inflammation (Bajaj et al., 2012b) (**Table 1**). However, these studies are all cross-sectional therefore can hardly provide little evidence for causal presumption, but there is still some value to be considered as some candidate biomarker, indicating that the pathogenesis of HE may be associated with the interaction between the immune system and microbiota (Arab et al., 2018).

**Table 1:** Microbiota differences in HE patients’ samples

Study	Study design	Samples	Microbiota changes	Potential affection	PMID
J. S. Bajaj, et al.	Cirrhotics with *vs*. without HE	Mucosal samples	Enterococcaceae ↑ - Enterococcus ↑	Intestinal infection and inflammatory response	21940902(32)
			Veillonellaceae ↑-Megasphaera ↑	N. A	
			Bifidobacteriaceae ↑ - Bifidobacterium ↑	Regulates synthesis of short-chain fatty acids and metabolism of oxalate	
			Lachnospiraceae ↓ - Roseburia ↓	Regulates bile acid metabolism	
Z. Zhang, et al.	MHE *vs*. no MHE	Fecal samples	Streptococcus salivarius ↑	N. A	23877352 (33)
J. S. Bajaj, et al.	OHE *vs.* no OHE	Fecal and sigmoid mucosal samples	Dorea ↑	Regulates bile acid metabolism	22821944 (97)
			Subdoligranulum ↑	N. A	
			Incertae sedis XIV ↑	N. A	
			Blautia ↑	Lipid metabolism	
			Roseburia ↑	Regulates bile acid metabolism	
			Faecalibacterium ↑	Intestinal infection and inflammatory response	
			Enterococcus ↓	Intestinal infection and inflammatory response	
			Burkholderia ↓	N. A	
			Proteus ↓	Intestinal infection and inflammatory response	
J. S. Bajaj, et al.	OHE *vs*. no OHE	Fecal samples	Enterobacteriaceae ↑	Intestinal infection, inflammatory response and cognitive	21940902 (98)
			Alcaligeneceae ↑	inflammatory response and Cognitive	
			Fusobacteriaceae ↑	SCFA metabolic	
			Ruminococcaceae ↓	N. A	
			Lachnospiraceae ↓	Intestinal infection and inflammatory response	

In a case report conducted by Kao, a 57-year-old man suffering from grades 1–2 HE with liver cirrhosis treated by FMT (Kao et al., 2016), despite a reduction in the relative abundance of Lachnospiraceae following FMT, showed an obvious improvement in cognition, suggesting that other microbial taxa and metabolic activity are involved. However, the result is from a single case report, so the universality of this conclusion remains in doubt. Interestingly, the inhibitory control test and Stroop test scores reverted to baseline by week 14, suggesting that the beneficial effect of fecal microbiota transplantation (FMT) is transient and that repeated therapy would be required to maintain the response (Kao et al., 2016). The results laterally demonstrate the correlation between gut microbiota and liver damage, indicating that persistent dysfunction of gut microbiota is one of the conditions for the development of HE and that liver function damage will facilitate this process. Although this study is the first report of treating HE with FMT and is well organized, it still has some unfortunate limitations, such as the lack of data on ammonia levels, as these parameters were not determined at a standardized time or in the fasting state, which suggests that we can reproduce such studies in animal models for more relevant data.

For changes in neuronal system, some very ancient studies of the end of the fifties of 20th century suggested the effect of oral neomycin on fecal flora so as to enterobatteriacae, such as *E. coli* and *Streptococcus faecalis*, with improvement in HE, especially in chronic neuropsychiatric disorder (Dawson et al., 1957; Summerskill, 1958). Ahluwalia conducted a study to define the individual contribution of specific gut bacterial taxa towards astrocytic and neuronal changes in brain function using multimodal magnetic resonance imaging (MRI) in patients with cirrhosis with or without HE (Ahluwalia et al., 2016). The study included 87 subjects with HE who underwent systemic inflammatory assessment, cognitive testing, stool microbiota analysis, and brain MRI analysis. Magnetic resonance spectroscopy (MRS) showed that the increased glutamate/glutamine and reduced myo-inositol and choline are hyperammonemia-associated astrocytic changes, while diffusion tensor imaging (DTI) demonstrated changes in neuronal integrity and edema. Linkages among cognition, MRI parameters, and gut microbiota were identified between the groups. Patients with HE had significantly worse cognitive performance, systemic inflammation, dysbiosis, and hyperammonemia than controls and cirrhotic patients without HE. Specific microbial families (autochthonous taxa negatively and Enterobacteriaceae positively) were correlated with MRS and hyperammonemia-associated astrocytic changes. However, Porphyromonadaceae was only correlated with neuronal changes on DTI without linkages to ammonia. We conclude that specific gut microbial taxa are related to the neuronal and astrocytic consequences of cirrhosis-associated brain dysfunction. Specific microbial families (autochthonous taxa negatively and Enterobacteriaceae positively) were correlated with the MRS and hyperammonemia-associated astrocytic changes (Ahluwalia et al., 2016), which suggests that some specific gut microbial taxa are related to the neuronal and astrocytic consequences of cirrhosis-associated brain dysfunction, indicating important roles in the pathogenesis of HE (Ahluwalia et al., 2016).

## Clinical and Therapeutic Strategies

Based on the comprehensive understanding of pathologic mechanisms and the role of gut microbiota in this process, the clinical implications of the basic research mentioned above are essential to promote the development of therapeutic interventions in HE. There is a growing body of epidemiological and experimental evidence to demonstrate the apparent effect of clinical therapies for HE that act through gut microbiota. Despite these promising results, the side effects and efficacy of therapies for HE are still being debated. Therefore, comprehensive identification and analysis of multiple treatments that function in modification of gut microbiota are required for further development of gut microbiota strategies for advanced therapies.

### Routine Therapy

The first serious discussions and analyses of gut microbiota-based therapeutics of HE emerged during the 2010s, and during the past 10 years, much more information has become available for the development of therapeutic strategies. Notably, some of these strategies are even routine treatments in HE, despite the limitations, which will help elucidate the impact of gut microbiota on HE.

#### Antibiotic Therapy

Antibiotics usually rapidly sterilize most bacteria. The introduction of antibiotics for non-infectious diseases such as HE is therefore a feasible option (Ianiro et al., 2016). Rifaximin is a semisynthetic antibiotic designed based on rifamycin, which has been shown to affect cognition in patients with HE by regulating the state of gut microbiota (Ng et al., 2019). Increasing evidence shows that rifaximin can significantly increase serum long-chain fatty acid and carbohydrate metabolic intermediate levels in patients with mild HE, which in turn affects serum pro-inflammatory cytokines and secondary bile acids to improve the structure of the gut microbiota and indices of gut immune function (Bajaj, 2016). Research further shows that changes in the metabolism of bacteria-produced agents, such as lipopolysaccharide and deoxycholic acid, caused by rifaximin contribute to maintaining normal gut microbiota levels (DuPont, 2016). In addition, rifaximin treatment significantly reduced the production of gut ammonia *via* the action of glutamine (Kang et al., 2016). Given the interactions between gut ammonia and gut barrier dysfunction, the detailed and proven molecular mechanisms could be related to gut microbiota (Peleman and Camilleri, 2016). Moreover, rifaximin reduced the diversity and abundance of ammonia-producing bacteria such as *Clostridium* and *Streptococcus*, a risk factor for HE, thus modifying the gut microbiota (Zuo et al., 2017). Notably, although the modulation of the microbiome by rifaximin in patients with HE was effective, there was no significant change in total gut bacterial load (Kang et al., 2016; Kawaguchi et al., 2019).

#### Prebiotics Supplementation

Despite indigestibility by the human, prebiotics have been attracting ever‐increasing attention owing to its regulatory effects of microbiota (Swennen et al., 2006). The particularly strong inhibition of intestinal absorption of ammonia in prebiotics was shown in research (Bosques-Padilla and Flores-Rendón, 2005), as it can cause massive colonic bacteria clearance through the production of hydrogen (Bongaerts et al., 2005). As a kind of prebiotic carbohydrate, lactulose has been applied in clinical practice since 1957 and is currently widely used to treat various intestinal disorders, including HE (Panesar and Kumari, 2011; Nath et al., 2018; Ruszkowski and Witkowski, 2019). The beneficial effects of lactulose may be associated with the effects on microbial metabolism (Tuohy et al., 2002; Ruiz-Aceituno et al., 2020), besides, studies undertaken so far provide conflicting evidence concerning the impact of lactulose on species richness in the gut microbiota (Sarangi et al., 2017; Tetz et al., 2017; Zhai et al., 2018). Therefore, exploring the interaction between lactulose and gut microbiota should be performed. Notably, lactulose can cause reversible qualitative and quantitative changes in fecal flora, which may explain the clinical efficacy of lactulose (Tuohy et al., 2002; Ferreira et al., 2019). A recent randomized controlled trial for patients with HE found showed obvious differences between actinomycetes, *Bacteroides*, *Pachybacteria*, and *Proteus* in the gut of excellent responders and non-responders to lactulose (Wang et al., 2019). This study provides prospective proposals for follow-up studies of lactulose intervention. Besides lactulose, there were other prebiotics to improve brain and liver function in HE patients. For example, herbal formulations exerted the hepaprotective effect by producing secondary metabolites (Tripathi and Tripathi, 2014). There is also evidence that treatment with fermentable fiber has possible therapeutic benefits in HE patients (Liu et al., 2004).

#### Probiotic Intervention

While the effects of intervention are generally positive, antibiotics cannot specifically alter the ecology of the gut microflora (Mahpour et al., 2020). Accompanied by unpredictable adverse effects, these treatments still have certain limitations (de Velde et al., 2018). In contrast, bacteriotherapy has gradually emerged because of its unique advantages. As early as 2011, probiotic interventions were used in bowel diseases such as colitis (Floch et al., 2011). By 2015, evidence from clinical trials showed that the effects of probiotics on gut microbiota have a significant impact on HE (Patel and DuPont, 2015). Further studies indicated that by preventing bacterial adhesion and translocation, probiotics can regulate blood metabolites related to the gut microbiota, such as cytokines, amino acids and vitamins (Qamar, 2015; Usami et al., 2015). Notably, the modification of the gut microflora using probiotics includes changes in the gut microbiota composition. *Clostridium* and *Bifidobacterium* were highly enriched, while enterococci and Enterobacteriaceae were significantly reduced, for example (Xia et al., 2018). In addition, probiotics cause changes in the gut microbiota by reducing the number of pathogenic bacteria, ammonia production and absorption as well as endotoxin levels, changing gut mucosal acidification, gut permeability, and the production of short-chain fatty acids, which play an essential role in improving the management of HE (Sharma and Singh, 2016). Recently, a decrease in C-reactive protein (P = 0.01), tumor necrosis factor (TNF) (P = 0.01), FABP-6 (P = 0.009), and claudin-3 (P = 0.002) with a sudden increase in neutrophil oxidation (P = 0.002) was observed in patients with HE following probiotic intervention (Roman et al., 2019); these factors are associated with enhanced immune adaptations that maintain homeostasis in the gut microbiota (Skonieczna-Zydecka et al., 2018). Thus, the results of this study provide further evidence to demonstrate a role for probiotics in the progression of HE. Given the few side effects and the costs of treatment, probiotic intervention has gradually become a common control measure for HE (Rivera-Flores et al., 2019). However, in terms of the degree of intervention, probiotics do not seem superior to lactulose or antibiotics in attaining remission in patients with HE (Cao et al., 2018). Therefore, further research on the relationship of prebiotics to gut microflora as well as more efficient treatments is still necessary.

The current medical treatment for HE is to regulate gut microbiota profiles and reduce ammonia production. Based on this principle and the effects of intervention programs, favorable effects can be observed from using multiple methods to improve HE (Schulz et al., 2019). More than 90% of patients with HE treated in prospective studies received a combination of rifaximin and lactulose. The clinical application of the combined intervention of probiotics and rifaximin is being actively pursued (Schulz et al., 2016). In addition, it has also been shown that combination of probiotic and prebiotic, also known as synbiotics, can reduce the activity of bacterial urease, which can reduce ammonia production, leading to improvement in HE patient outcomes (Sekhar et al., 2013; Patel and DuPont, 2015). During the past several years, multiple clinical trials and case reports have demonstrated the efficacy of synbiotics play a pivotal role in the treatment of HE. For example, Liu et al. (2004) describe the effect of synbiotic treatment in HE patients (97 study participants). Specifically, after receiving synbiotic treatment, the fecal content of non-urease-producing Lactobacillus species increased significantly, which correlated with a rapid reduction in plasma ammonia levels. However, as mentioned above, all methods have attendant limitations, which have barely been addressed. Therefore, the development of new efficient therapies to directly block or slow disease progression is very important (Weir and Reddy, 2020).

#### Dietary Intervention

The composition of the gut microbiota within the human body is strongly associated with dietary habits (Rinninella et al., 2019). Given the important role of gut microbiota in the pathogenesis of HE, dietary change has been recommended as a treatment strategy with the potential to alleviate symptoms in HE (Campion et al., 2019). A recent cross-sectional study in 275 participants shows that lower protein and animal fat intakes in the diets of persons is associated with the increased risk of HE, which is accompanied by differences of gut microbiota, such as Prevotellaceae, Ruminococcaceae and Lachnospiraceae (Bajaj et al., 2020). In addition, it was demonstrated that different kinds of proteins in the diet may contribute towards an improved treatment outcome of HE, branched-chain amino acid intake equally contribute to decrease risk of recurrence in HE (Amodio et al., 2014). Considering the beneficial effects of prebiotics on gut microbiota, it has been convincingly demonstrated that fiber-rich diets can induce beneficial changes on gut microbiota in healthy people, including reduction of *Bacteroides* spp., Clostridium cluster XIVa bacteria and Enterobacteriaceae, yet their health impact in HE patients is unclear (Merli et al., 2016).

### Novel Therapy

As we are increasingly improving our knowledge about the intestinal microbiota, the limitations of routine therapies in HE, such as the abuse of antibiotics, have led to a dramatic increase in microorganism resistance and have presented increasing challenges to the discovery and development of novel therapies (Laffin et al., 2017).

#### Fecal Microbiota Transplantation

**Figure 4:**
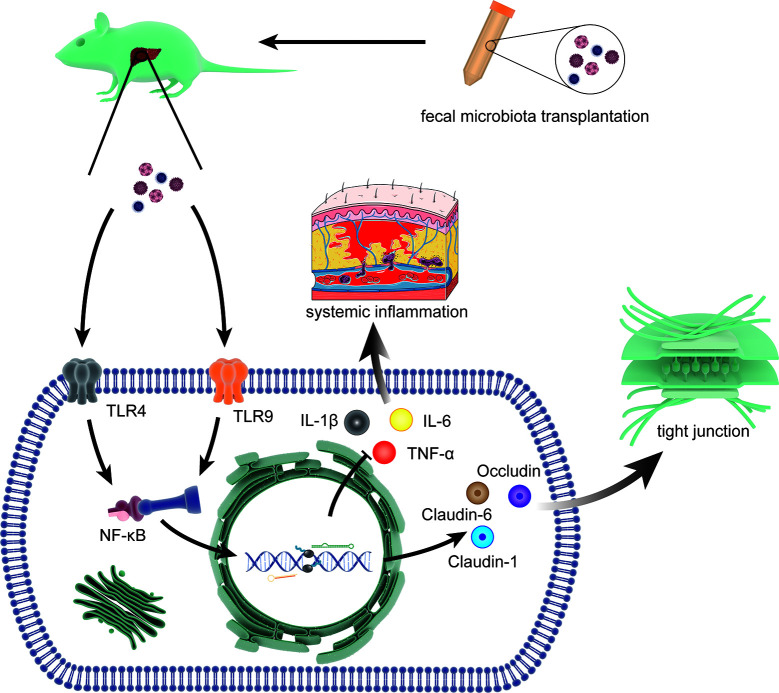
The molecular mechanisms involved in FMT intervention. The process includes limiting the systemic inflammatory response by reducing the expression of interleukin (IL)-1β, IL-6 and TNF-α through toll-like receptor (TLR)4 and TLR9 (two effective inflammatory mediators) *via* NF-*κ*B signal and restoring the tight junction proteins (Claudin-1, Claudin-6, and Occludin) that was lost due to HE.

Recently, Liu, R. demonstrated that fecal microbial colonization from patients with HE is associated with neuroinflammation, microglial activation, and ecological disorders by experiments involving colonization in germ-free (GF) mice. This result implied a link among HE, gut microbiota, and fecal microbes (Liu et al., 2020). FMT refers to the transfer of feces from healthy donors to patients with disordered gut microbes. The concept of FMT emerged in the 14th century, but the contribution of FMT to HE has only recently begun to be elucidated (Vindigni and Surawicz, 2017). In 2015, Shen, T. C. et al. first inoculated microbiota in mice with experimental liver injury to reduce the production of ammonia in the gut and the risk of neurotoxicity and encephalopathy associated with hyperammonemia (Shen et al., 2015). Interestingly, a related report suggested the application of FMT in the intervention of HE in that same year (Kang et al., 2015). In 2016, the first case report of FMT for the treatment of HE demonstrated that serial FMT can improve cognitive function in patients with mild HE (Kao et al., 2016). Based on these observations, the first randomized clinical trial in 2017 proved that reasonable donor selection of FMT can reduce the length of hospital stay in patients with HE and improve their cognitive ability and quality of life (Bajaj et al., 2017). Further studies with mouse models of HE indicated that FMT can prevent damage to the gut mucosal immune barrier function and liver necrosis and reduce serum levels of ammonia by enhancing hepatic clearance and gut epithelial permeability (Wang et al., 2017) (**Figure 4**). The effect of FMT on the gut microbiota is mainly due to the increase in Ruminococcaceae and Bifidobacteriaceae and the decrease in Streptococcaceae and Veillonellaceae. This change was accompanied by an increase in E-cadherin (P = 0.03) and defensin alpha 5 (P = 0.03) and a decrease in interleukin-6 (P = 0.02) and serum lipopolysaccharide binding protein (LBP) (P = 0.009) (Bajaj et al., 2019). In 2018, the effect of FMT on regulation of HE was included in the guidelines for use of FMT, increasing the interventions of HE (Mullish et al., 2018). Although the potential of FMT to alter the course of HE is promising, the lack of basic research has led to a lack of understanding about the limitations of FMT (Millan et al., 2017). In addition, infections with microbial pathogens due to lack of donor screening have occurred in multiple retrospective case series (DeFilipp et al., 2019). Therefore, FMT is currently only used in experimental settings and is not a standard treatment.

In addition, potential therapeutic targets in HE have been discovered, including ammonia scavengers (ornithine phenylacetate) (Alsahhar and Rahimi, 2019), TNP-2092 (a multitargeting drug conjugate with extremely low propensity for resistance development) and gut mannitol (enteral mannitol administration) (Yuan et al., 2020), which are related to gut microbiota. Therefore, further research on the links between gut microbiota and HE may provide prospective guidance for the development of interventions for HE. Based on these results, it is interesting to note that non-pharmacological measures that regulate the gut microbiota, such as exercise and nutritional supplements, may play a major role in modulating the course of HE (Rowland et al., 2018).

## Conclusion

This review aimed to provide the first systematic account of the influence of gut microbiota in hepatic encephalopathy as well as to assess the effects of therapeutic strategies. The findings clearly indicate the regulation of HE by gut microbiota through a complex signaling network, while some factors emerged as reliable predictors and prognostic biomarkers. The generalizability of this review is mainly limited by its focus solely on cellular and molecular factors; genetic factors were unable to be analyzed, as this subject has not been examined in this field. Notwithstanding these limitations, this work offers valuable insights into the pathogenesis and management strategies of HE from the gut microbiota perspective. Further research on the genetic background of HE related to the gut microbiota is therefore an essential next step in improving the pathogenic genetic and molecular networks controlling HE.

## Author Contributions

All authors listed have made a substantial, direct, and intellectual contribution to the work and approved it for publication.

## Funding

This work was supported by funding from the National Natural Science Foundation of China [grant numbers: 81770306, 81971820], the Province Natural Science Foundation of Hunan [grant numbers: 2018JJ2547].

## Conflict of Interest

The authors declare that the research was conducted in the absence of any commercial or financial relationships that could be construed as a potential conflict of interest.

## Abbreviation

HE, hepatic encephalopathy; SCFA, short-chain fatty acids; FMT, fecal microbiota transplantation; MRI, magnetic resonance imaging; MRS, magnetic resonance spectroscopy; DTI, diffusion tensor imaging; TNF, tumor necrosis factor; LBP, lipopolysaccharide binding protein; TLR, toll-like receptor; IL, interleukin.
